# Lipopolysaccharide perception leads to dynamic alterations in the microtranscriptome of *Arabidopsis thaliana* cells and leaf tissues

**DOI:** 10.1186/s12870-015-0465-x

**Published:** 2015-03-07

**Authors:** Arnaud T Djami-Tchatchou, Ian A Dubery

**Affiliations:** Department of Biochemistry, University of Johannesburg, P.O. Box 524, Auckland Park, 2006 South Africa

**Keywords:** *Arabidopsis thaliana*, Lipopolysaccharides, miRNAs, Illumina sequencing, Expression profiles

## Abstract

**Background:**

MicroRNAs (miRNAs) are non-coding RNA molecules which have recently emerged as important gene regulators in plants and their gene expression analysis is becoming increasingly important. miRNAs regulate gene expression at the post-transcriptional level by translational repression or target degradation of specific mRNAs and gene silencing. In order to profile the microtranscriptome of *Arabidopsis thaliana* leaf and callus tissues in response to bacterial lipopolysaccharide (LPS), small RNA libraries were constructed at 0 and 3 h post induction with LPS and sequenced by Illumina sequencing technology.

**Results:**

Differential regulation of subset of miRNAs in response to LPS treament was observed. Small RNA reads were mapped to the miRNA database and 358 miRNAs belonging to 49 miRNA families in the callus tissues and 272 miRNAs belonging to 40 miRNA families in the leaf tissues were identified. Moreover, target genes for all the identified miRNAs families in the leaf tissues and 44 of the 49 miRNAs families in the callus tissues were predicted. The sequencing analysis showed that in both callus and leaf tissues, various stress regulated-miRNAs were differentially expressed and real time PCR validated the expression profile of miR156, miR158, miR159, miR169, miR393, miR398, miR399 and miR408 along with their target genes.

**Conclusion:**

*A. thaliana* callus and leaf callus tissues respond to LPS as a microbe-associated molecular pattern molecule through dynamic changes to the microtranscriptome associated with differential transcriptional regulation in support of immunity and basal resistance.

**Electronic supplementary material:**

The online version of this article (doi:10.1186/s12870-015-0465-x) contains supplementary material, which is available to authorized users.

## Background

The first plant microRNAs (miRNAs) were described by isolating, cloning, and sequencing small RNA populations in *Arabidopsis thaliana* and later in other species [1]. In Arabidopsis and rice, miRNAs and their targets have been extensively studied [2,3].

miRNAs are a class non-coding, sequence-specific and *trans*-acting endogenous small RNAs that play very important roles in post-transcriptional gene regulation through degradation of target mRNAs or by translational repression of targeted genes [4,5]. Currently, more and more investigations in functional analysis of conserved miRNAs reveal their involvement in multiple biological and metabolic processes in plants, including induced responses towards abiotic – and biotic stressors, by modulating the expression of their endogenous target genes [6-10].

RNA polymerase II transcribes miRNAs into long primary transcripts (pri-miRNAs) that are cut into miRNA precursors (pre-miRNAs) with typical hairpin structures, capped with a specially modified nucleotide at the 5’ end and polyadenylated with multiple adenosines [6,11]. The pre-miRNA hairpin is cleaved to generate the mature miRNAs from the stem portion of the single stranded stem-loop precursor by the complex containing the nuclear RNase III enzyme and the ribonuclease III-like enzyme Dicer (DCL1) [12]. The resulting mature miRNA is inserted into the RNA-induced silencing complex (RISC) that contain argonaute proteins. Finally the mature miRNA guides the RISC to complementary mRNA targets and the RISC inhibits translation elongation or triggers the degradation of target mRNA [13].

Many of the target genes of miRNAs identified in plants, either computationally (comparative genomics) or experimentally (cloning and deep sequencing, northern blotting, and/or quantitative real time PCR), encode regulatory proteins, indicative of the function of miRNAs as important regulators for gene expression [5,14-16]. The discovery of the ability of miRNAs to regulate gene expression suggests that this class of non-coding RNAs represent one of the more abundant classes of gene regulatory molecules in plants and possibly affect the output of many protein-coding genes [8,14,15,17].

Experimental studies in Arabidopsis and other plants have shown that abiotic and biotic stresses induce differential expression of a set of miRNAs such as: miR156, miR159, miR165, miR167, miR168, miR169, miR319, miR393, miR395, miR396, miR398, miR399, and miR402 [7,18-23]. Some of their identified target genes were involved in signaling pathways and regulation of gene expression and transcription associated with the stress conditions [7,10]. Recent evidence shows that miRNAs are substantially implicated as molecular regulators of plant immune – and defense responses [8,24-27].

Plants exhibit a sophisticated molecular system for recognition of microbe-associated molecular pattern (MAMP) molecules and undergoes a massive reprogramming of the transcriptome upon perception of MAMPs [28], leading to MAMP-triggered immunity (MTI). One of the prototypic model MAMPs used as potential inducers of plant defense responses is bacterial lipopolysaccharide (LPS), a major component of the outer membrane of Gram-negative bacteria [28-31]. Perception of LPS leads to the activation of an array of defense genes in *A. thaliana* in support of innate immunity and MTI [32,33].

High-throughput (H-T) sequencing technologies have provided a powerful tool for enhancing miRNA discovering and target identification in plants [6,10,34,35]. With its massively parallel throughput, this has revolutionized the analysis of microtranscriptomes for low-cost and high quality. Millions of miRNAs sequences can be generated and used directly for identification and profiling expression level of miRNAs with a possibility to compare the expression profiles of two or more samples [36].

miRNAs have emerged as a potential means to obtain insight into the nature of complex regulatory networks operating during plant-microbe interactions. In this study we employed Illumina sequencing technology to gain a global picture of the expression profiles of miRNAs in undifferentiated cultured *A. thaliana* cells following the induction of defense responses using LPS. The findings were subsequently extended to also include differentiated leaf tissue. This is the first microtranscriptome study, using LPS as a MAMP, to identify miRNAs differentially expressed in *A. thaliana* cells and leaf tissues and their target genes. LPS is only one of a cocktail of MAMPs that a plant might perceive upon attempted bacterial infection and as such its responses are expected to be more specific compared to the responses elicited by a combination of different MAMPs.

## Results

### miRNAs isolation and sequencing

**Figure 1:**
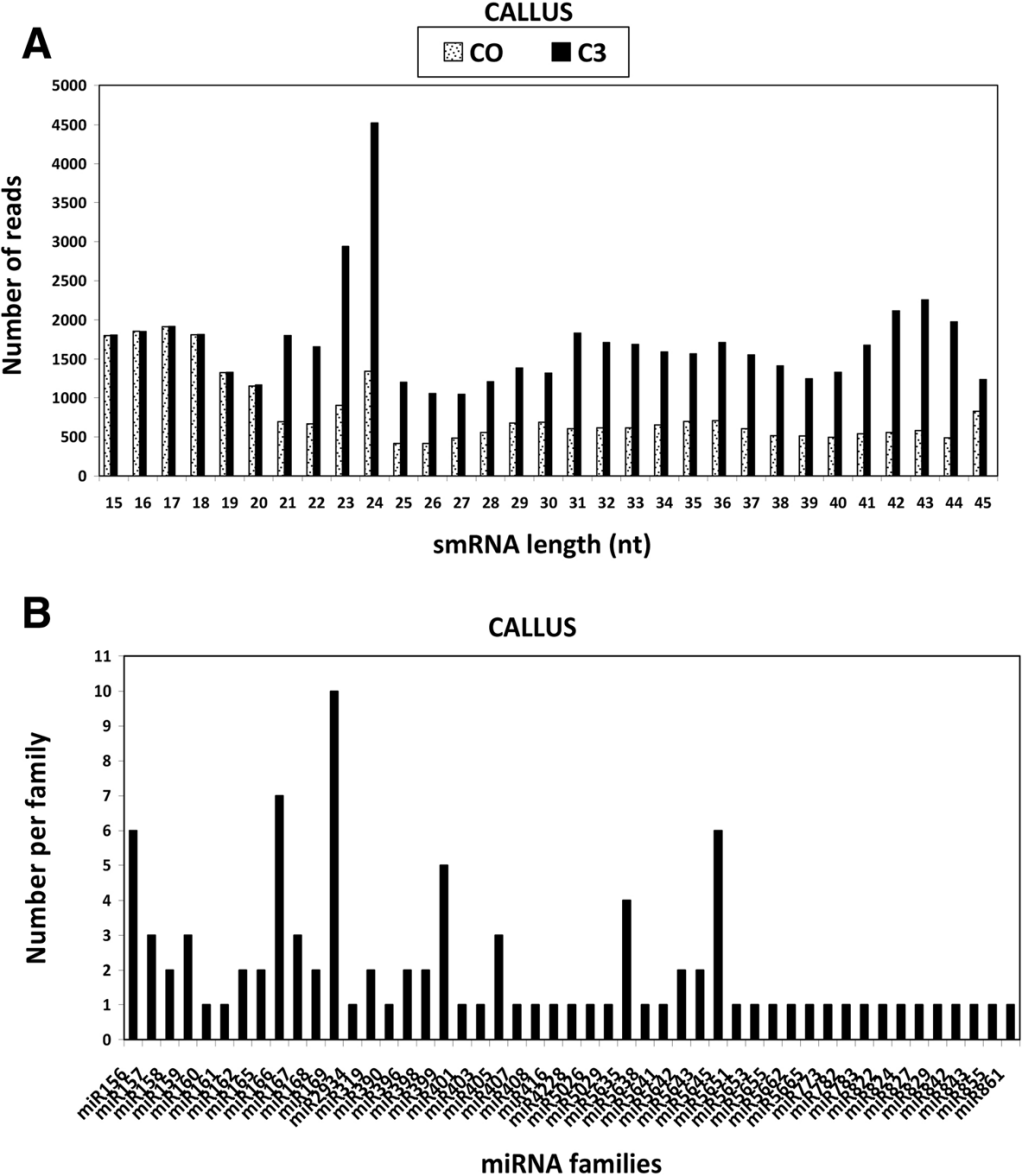
**Size distribution of** ***A. thaliana*** **small RNAs from callus tissue. A**: Size range of identified small RNAs; **B**: Identified miRNA families (49 miRNA families).

**Figure 2:**
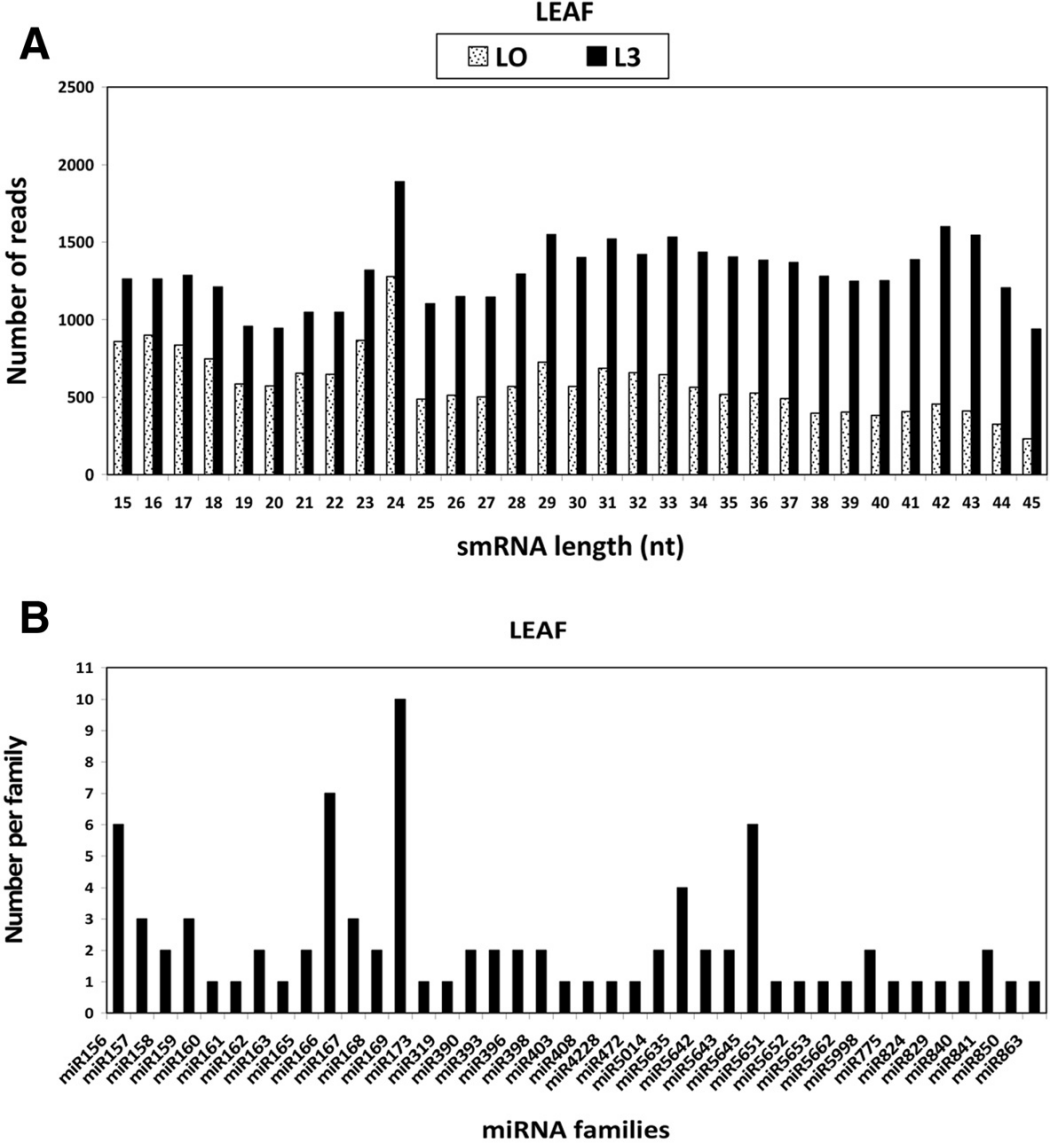
**Size distribution of** ***A. thaliana*** **small RNAs in leaf tissue. A**: Size range of identified small RNA; **B**: Identified miRNA families (40 miRNA families).

In order to profile the composition and expression of Arabidopsis miRNAs in response to LPS treatments we isolated miRNA from *A. thaliana* callus and leaf tissues after 0 and 3 h post treatment. Four small RNA libraries (2 from each type of plant material) were constructed and sequenced using the Illumina H-T sequencing technology. A total of 7 994 362 raw reads was generated for the callus tissues (control, C0 and treated, C3 samples) and the leaves (control, L0 and treated, L3 samples). After quality control and adapter trimming, a total 1 557 720 high quality clean reads was obtained (Additional file 1: Table S2). Following sequence filtering on length (reads < 15 nucleotides or > 55 nucleotides discarded) 131 042 reads were obtained which were then subjected to analyse their length distribution. The small RNAs were in the range of 15 to 45 nucleotides in both callus and leaf libraries (Figures 1A and 2A). In terms of total sequence abundance, the class of small RNA with 24 nucleotides length was shown to be the most abundant in both tissues. The total number of small RNA sequences identified from the treated libraries was larger compared with the control libraries for both callus and leaf tissues.

### miRNA identified in *A. thaliana* leaf and callus tissues untreated and treated with LPS

The small RNA sequences from the control and treated samples were mapped to the *A. thaliana* genome and miRBase release 20.0 for miRNA identification. Only small RNA reads that perfectly matched known *A. thaliana* miRNA from miRBase were selected. Sequence similarity search enabled us to identify in callus tissues 358 miRNAs belonging to 49 miRNA families (Table 1) and in the leaf tissues 272 miRNAs belonging to 40 miRNA families from Arabidopsis (Table 2). The number of representative miRNA members per family varied from 1 to a maximum of 10 per family (Figures 1B and 2B). All the 630 miRNAs identified in this study represent highly conserved plant miRNAs.

**Table 1:** **Expression profiling of miRNA differentially expressed in** ***A. thaliana*** **callus tissues in response to LPS elicitation (C0 = untreated control, C3 = treated for 3 h)**

**miRNA**	**Mature sequence**	**Length**	**CO (count)**	**C3 (count)**	**C3/C0**	**Fold-change log**_**2**_ **C3/C0**
miR156	TGACAGAAGAGAGTGAGCAC	20	1	15	15	3.9 ↑
miR159	TTTGGATTGAAGGGAGCTCTA	21	10	41	4.1	2.0 ↑
miR160	TGCCTGGCTCCCTGTATGCCA	21	0	1		NA*
miR162	TCGATAAACCTCTGCATCCAG	21	0	2		NA*
miR165	TCGGACCAGGCTTCATCCCCC	21	1	3	3	1.6 ↑
miR166	TTCGGACCAGGCTTCATTCCCC	22	10	26	2.6	1.4 ↑
miR167	GATCATGTTCGCAGTTTCACC	21	6	2	0.33	−1.6 ↓
miR169	CTGGCAAGTTGACCTTGGCTCTGC	24	0	4		NA*
miR2934	TCTTTCTGCAAACGCCTTGGA	21	1	0		NA*
miR319	TTGGACTGAAGGGAGCTCCTTC	22	0	2		NA*
miR396	TTCCACAGCTTTCTTGAACTG	21	73	171	2.34	1.2 ↑
miR398	GGGTTGATATGAGAACACACG	21	1	4	4	2 ↑
miR401	ACAATGGAGATTAGGAGACATTTT	24	1	7	7	2.8 ↑
miR403	GGATTAGATTCACGCACAAACTC	23	0	1		NA*
miR405	AAATGAGTTATGGGTTAGACCCGT	24	4	13	3.25	1.7 ↑
miR407	GGGGAAAAATGTCAAAAAAATCGC	24	0	1		NA*
miR408	TGCACTGCCTCTTCCCTGGCT	21	0	12		NA*
miR5026	AAAGTTAGTAACTCAAAGGCTCGT	24	3	1	0.33	−1.6 ↓
miR5029	AAUGAGAGAGAACACUGCAAA	24	0	1		NA*
miR5635	ATTTAATACCTGAACTTTCAAAGA	24	6	20	3.33	1.7 ↑
miR5638	TCCACACTAGTGTAACGACAGTG	23	0	1		NA*
miR5641	CATGGAGGAGATATTTGGTAA	21	0	1		NA*
miR5642	CAAGAACATCTTCGTTACGGAT	17	58	119	2.05	1.1 ↑
miR5643	AGAAGACACAGAGACAAAGACTCA	24	13	34	2.62	1.4 ↑
miR5651	TCGTTACTATTTGAACCGCACC	22	0	1		NA*
miR5655	CTTTTCCTCCTCCTCCACCACC	22	1	0		NA*
miR5662	AGAGGAAAATATAGAGATCACCAT	24	0	2		NA*
miR773a	TTTGCTTCCAGCTTTTGTC	19	1	0		NA*
miR782	TCTTTCTGCAAACGCCT	17	1	0		NA*
miR783	CAAAAGATCTGGTGATGAAGTTGA	24	0	1		NA*
miR822	GCGGGAAGCATTTGCACATGTT	22	0	1		NA*
miR824	CCTTCTCATCGATGGTCTAGA	21	0	2		NA*
miR829	AGCTCTGATACCAAATGA	18	0	4		NA*
miR842	CATGGTCAGATCCGTCATCCC	21	0	1		NA*
miR855	AAACTCGAAAGCGTCTAGGACTTT	24	0	2		NA*

NA*: Relative change was not calculated as they contained 0 reads in one sample; Log_2_ ratio of normalized miRNA expression in stress and control libraries.

C0: control, C3: treated condition; ↑ and ↓: up- and down regulated responses.

**Table 2:** **Expression profiling of miRNA differentially expressed in** ***A. thaliana*** **leaf tissues in response to LPS elicitation (L0 = untreated control, L3 = treated for 3 h)**

**miRNA**	**Mature sequence**	**Length**	**LO (count)**	**L3 (count)**	**L3/L0**	**Fold-change log**_**2**_ **L3/L0**
miR158	TCCCAAATGTAGACAAAGCA	20	95	45	0.47	−1.1 ↓
miR160	TGCCTGGCTCCCTGTATGCCA	21	0	3		NA*
miR167	TGAAGCTGCCAGCATGATCTA	21	13	59	4.54	2.2 ↑
miR169	TAGCCAAGGATGACTTGCCT	20	0	9		NA*
miR173	TTCGCTTGCAGAGAGAAATCAC	22	1	0		NA*
miR393	ATCATGCGATCTCTTTGGAT	20	3	0		NA*
miR396	TTCCACAGCTTTCTTGAACT	20	314	140	0.45	−1.2 ↓
miR398	TGTGTTCTCAGGTCACCCCTG	21	2	6	3	1.6 ↑
miR408	ATGCACTGCCTCTTCCCTGGC	21	0	1		NA*
miR472	TTTTTCCTACTCCGCCCATACC	22	0	2		NA*
miR5014	TTTTCACTGTTTGATTCGTACACT	24	1	0		NA*
miR5635	GTATAAAACGATCATTTCAAGAGT	24	2	5	2.5	1.3 ↑
miR5642	TCGACACCTTGCGGCTAGGAAC	22	15	41	2.73	1.4 ↑
miR5651	GTTCGATCACCATTCGGAGCCT	22	1	0		NA*
miR5653	AACTCAACCCATGAACCCTAATGA	24	1	0		NA*
miR5662	ATTTTAGAGGTGACTAT	17	0	1		NA*
miR5998	TGTTTTGTTTTGTGATGTTGGAACAAAT	28	0	1		NA*
miR775	TTCGATGTCTAGCAGTGCCT	20	0	2		NA*
miR829	AGCTCTGATACCAAATGA	18	3	0		NA*
miR840	TTGTTTAGGTCCCTTAGTTTCT	22	0	1		NA*
miR850	AAGATCCGGACTACAACAAAGC	22	1	0		NA*

NA*: Relative change was not calculated as they contained 0 reads in one sample; Log_2_ ratio of normalized miRNA expression in stress and control libraries.

L0: control, L3: treated condition; ↑ and ↓: up- and down regulated responses.

### Predicted target genes of identified miRNAs

Due to the importance of miRNA in regulating gene expression and for better understanding of their biological mechanisms by which *A. thaliana* responds to LPS, the putative target genes of miRNAs were identified by aligning miRNA sequences with the miRBase using the web-based psRNATarget program [35,37]. In the leaf tissues target genes were identified for all the identified miRNAs and in the callus tissues the same, except for miR5638, miR773, miR782 and miR843 (Tables 3 and 4). In *A. thaliana*, many of the miRNA – mRNA interactions have been experimentally validated. In total about 86 targets genes were predicted among which most of them encode transcription factors (TFs) targeted by miR156, miR159, miR165, miR166, miR169, miR319, miR408, miR829, miR2934, miR5029 and miR5642. The knowledge of the target genes’ identified functions informed on the subsequent miRNA studies.

**Table 3:** **Target prediction of the miRNAs differentially expressed in** ***A. thaliana*** **callus tissues in response to LPS elicitation**

**miRNA**	**Target Acc**	**miRNA Sequence**	**Target aligned fragment**	**Expectation**	**Target start**	**Target end**	**Target description**
miR156	AT5G43270.1	TGACAGAAGAGAGTGAGCAC	GUGCUCUCUCUCUUCUGUCA	1.0	1188	1207	Squamosa promoter binding protein-like 2
miR158	AT1G64100.1	TCCCAAATGTAGACAAAGCA	UUCUUUGUCUACAUUUGGCA	2.5	477	496	Pentatricopeptide (PPR) repeat-containing
miR159	AT2G32460.1	TTTGGATTGAAGGGAGCTCTA	UAGAGCUUCCUUCAAACCAAA	2.0	1004	1024	Myb domain protein 101, ACC synthase
miR160	AT2G28350.1	TGCCTGGCTCCCTGTATGCCA	GGAAUACAGGGAGCCAGGCA	1.0	1330	1349	Auxin response factor 10
miR162	AT1G01040.1	TCGATAAACCTCTGCATCCAG	CUGGAUGCAGAGGUAUUAUCGA	2.0	3422	3443	DICER-LIKE1
miR165	AT2G34710.1	TCGGACCAGGCTTCATCCCCC	UGGGAUGAAGCCUGGUCCGG	1.5	871	890	Homeobox-leucine zipper family protein
miR166	AT2G34710.1	TTCGGACCAGGCTTCATTCCCC	GGGAUGAAGCCUGGUCCGGA	1.0	872	891	Homeobox-leucine zipper family
miR167	AT1G67120.1	GATCATGTTCGCAGTTTCACC	GGUGAAACUGCGUCACAUGAUC	3.0	1909	1930	Transcription factor binding; ARF6
mIR169	AT1G17590.1	TAGCCAAGGATGACTTGCCT	AGGGAAGUCAUCCUUGGCUG	1.5	1233	1252	CCAAT-binding TF (CBF-B/NF-YA)
miR2934	AT1G49770.1	TCTTTCTGCAAACGCCTTGGA	CCAAGGCUUAUGCAGAGAGA	2.5	992	1011	bHLH DNA-binding superfamily protein
miR319	AT3G15030.1	TTGGACTGAAGGGAGCTCCTTC	GAGGGGUCCCCUUCAGUCCAG	2.5	1476	1496	TCP family transcription factor 4
miR396	AT2G36400.1	TTCCACAGCTTTCTTGAACTG	CCGUUCAAGAAAGCCUGUGGAA	2.0	746	767	Growth-regulating factor 4
miR398	AT4G27050.2	GGGTTGATATGAGAACACACG	GUGUGUUUUUAUGUCAAUCU	2.5	154	173	F-box/RNI-like superfamily protein
miR399	AT2G33770.1	TGCCAAAGGAGAGTTGCCCTG	AGGGCAAAUCUUCUUUGGCA	1.5	608	627	Ubiquitin-protein ligase
miR401	AT5G34863.1	ACAATGGAGATTAGGAGACATTTT	AAAAUGUCUCCUUAUCUCCAUUGU	1.0	329	352	Transposable element gene
miR403	AT1G31280.1	GGATTAGATTCACGCACAAACTC	GAGUUUGUGCGUGAAUCUAAUUG	1.5	3223	3245	AGO2 | Argonaute family protein
miR405	AT1G27880.1	AAATGAGTTATGGGTTAGACCCGT	GUCUGGUCCAAGACUCAUUU	3.0	2633	2653	DEAD/DEAH box RNA helicase family protein
miR407	AT3G20220.1	GGGGAAAAATGTCAAAAAAATCGC	UGAUUUUUUUGAUAUCUUUCUUU	3.0	197	219	SAUR-like auxin-responsive protein family
miR408	AT2G02850.1	TGCACTGCCTCTTCCCTGGCT	CCAAGGGAAGAGGCAGUGCA	2.0	97	116	Plantacyanin
miR5026	AT3G62340.1	AAAGTTAGTAACTCAAAGGCTCGT	AGCUUUUGAGUUUUUAACUUC	3.0	166	186	WRKY68, AtWRKY68 | WRKY family TF
miR5029	AT4G04570.1	AAUGAGAGAGAACACUGCAAA	UUGUGGUGUUUACUUUCAUU	3.0	964	983	Cysteine-rich RLK
miR5635	AT1G20230.1	ATTTAATACCTGAACTTTCAAAGA	UGGAAAGUUCAGGUAUUGAGG	3.0	623	643	Pentatricopeptide repeat (PPR)
miR5638		TCCACACTAGTGTAACGACAGTG					Unknown
miR5641	AT3G43571.1	CATGGAGGAGATATTTGGTAA	UUAUCUAAUAUUUCUUCCAUG	2.5	528	548	Transposable element gene
miR5642	AT5G04010.1	CAAGAACATCTTCGTTACGGAT	AGUUUUAAUGAAGAUGUUCUUG	2.5	754	775	F-box family protein
miR5643	AT1G71840.1	AGAAGACACAGAGACAAAGACTCA	GGGUUUUUGUACUUUGUGUCUUCU	3.0	71	94	Transducin WD-40 repeat family protein
miR5651	AT1G43260.1	TCGTTACTATTTGAACCGCACC	UGUUGUUCAAGUAGUAACGA	2.0	504	523	hAT transposon superfamily protein
miR5655	AT5G02480.1	CTTTTCCTCCTCCTCCACCACC	GGUGGUGGAGGAGGAGGAGGAG	1.0	992	1013	HSP20-like chaperones superfamily protein
miR5662	AT5G60410.1	AGAGGAAAATATAGAGATCACCAT	UGGUUGUCUUUUUAUUUUCCUCU	3.0	10	32	DNA-binding protein: MIZ/SP-RING Zinc finger, PHD-finger
miR773	AT5G64270.1	TTTGCTTCCAGCTTTTGTC	AGACGGAAGAUGGAAGCAGA		172	191	Splicing factor, putative
miR782	AT3G29152.1	TCTTTCTGCAAACGCCT	AGAACACCAAACGUGUUUGU		260	279	Protease inhibitor/seed storage/LTP family protein
miR783	AT5G43530.1	CAAAAGATCTGGTGATGAAGTTGA	UGGACUUCAUUUUUGGAUCUUUUG	3.0	3406	3429	Helicase protein with RING/U-box domain
miR822	AT5G02330.1	GCGGGAAGCATTTGCACATGTT	AGCAUGUGCAAAUGCUUCUCGC	0.5	1254	1275	Cysteine/Histidine-rich C1 domain family protein
miR824	AT3G08870.1	CCTTCTCATCGATGGTCTAGA	UUUAGGCCAUCGAUGAGAAUG	2.5	1873	1893	Concanavalin A-like lectin protein kinase protein
miR829	AT5G18560.1	AGCTCTGATACCAAATGA	UUACCUUGAAGUUUUGAUUUG	1.5	1284	1304	AP2 domain-containing TF/ ethylene response factor
mIR842	AT1G60130.1	CATGGTCAGATCCGTCATCCC	GGGAUGAUGGAUCCGACCAUG	1.5	86	106	Mannose-binding lectin superfamily protein
miR855	AT5G02490.1	AAACTCGAAAGCGTCTAGGACTTT	ACUUUUUCUUAGCUUUUUCU	3	47	66	Heat shock cognate 70 kDa protein 2 (HSC70-2) (HSP70-2)

**Expectation**: value assigned to the alignment of the mature miRNA and the target. The value ranges from 0 (perfect alignment) to 5); **Target start**: the base position where the annealing with the miRNA starts; **Target end**: the base position where the annealing with the miRNA ends.

**Table 4:** **Target prediction of the miRNA differentially expressed in** ***A. thaliana*** **leaf tissues in response to LPS elicitation**

**miRNA**	**Target Acc**	**miRNA Sequence**	**Target aligned fragment**	**Expectation**	**Target start**	**Target end**	**Target description**
miR156a	AT1G27360.1	TGACAGAAGAGAGTGAGCACC	GUGCUCUCUCUCUUCUGUCA	2.5	1253	1272	Squamosa promoter-binding-like protein
miR158	AT1G64100.1	TCCCAAATGTAGACAAAGCA	UUCUUUGUCUACAUUUGGCA	2.5	477	496	Pentatricopeptide (PPR) repeat protein
miR159	AT2G32460.2	TTTGGATTGAAGGGAGCTCTA	UAGAGCUUCCUUCAAACCAAA	2.0	972	992	Myb domain protein 101, ACC synthase
miR160	AT1G77850.1	TGCCTGGCTCCCTGTATGCCA	CCUGAUGUAAUCACUUUCAA	0.5	1409	1429	ARF10 (Auxin response factor 10)
miR167	AT1G30330.1	TGAAGCTGCCAGCATGATCTA	UGAUCAUUCUGGCAGCUUUG	2.5	3276	3296	ARF6 (Auxin response factor 6)
miR169	AT1G17590.1	TAGCCAAGGATGACTTGCCT	AGGGAAGUCAUCCUUGGCUG	1.5	1233	1252	Nuclear factor Y, subunit A8
miR173	AT2G27400.1	TTCGCTTGCAGAGAGAAATCAC	UCGUUUCCCUCUGUAAGCGAA	1.5	367	388	Trans-acting siRNA1a primary transcript
miR393	AT3G59740.1	ATCATGCGATCTCTTTGGAT	AUCCAAAGAAGUCGUAUGAU	2.0	794	813	Concanavalin A-like lectin protein kinase
miR396	AT3G52910.1	TTCCACAGCTTTCTTGAACTG	CCGUUCAAGAAAGCCUGUGGAA	2.0	742	763	Growth-regulating factor 4
miR398	AT1G08830.1	TGTGTTCTCAGGTCACCCCTG	UGUGUUCUCAGGUCACCCCUG	2.0	86	106	Copper superoxide dismutases
miR408	AT2G02850.1	ATGCACTGCCTCTTCCCTGGC	CAAAGGGAAGAGGCAGUGCAU	1.0	98	117	Plantacyanin
miR472	AT1G51480.1	TTTTTCCTACTCCGCCCATACC	GGUAUGGGGGGAGUAGGAAAAA	1.0	799	820	Disease resistance protein (CC-NBS-LRR)
miR5014	AT1G05840.1	TTTTCACTGTTTGATTCGTACACT	GACGAAUCAGACAGUGGAAA	2.0	519	538	Eukaryotic aspartyl protease family protein
miR5635	AT3G11430.1	GTATAAAACGATCATTTCAAGAGT	UUUGAGAUGAUUUUUUUAUAU	2.5	1779	1799	Glycerol-3-phosphate acyltransferase 5
miR5642	AT1G21080.2	TCGACACCTTGCGGCTAGGAAC	GUUCUUAGCUGAAAAGUGUCGA	3.0	54	75	DNAJ heat shock N-terminal domain-protein
miR5651	AT1G72200.1	GTTCGATCACCATTCGGAGCCT	GAUCCGAACGGUGAUCGGAU	3.0	809	828	RING/U-box superfamily protein
miR5653	AT4G02200.1	AACTCAACCCATGAACCCTAATGA	UCAGUCGGGUUCAUGUAUUGAGUU	3.0	144	167	Drought-responsive family protein
miR5662	NP10427100	ATTTTAGAGGTGACTAT		1.5	508	529	Pentatricopeptide repeat (PPR) protein
miR5998	AT3G46230.1	TGTTTTGTTTTGTGATGTTGGAACAAAT	GUUCUAGCAAAACAAAACAAAACA	3.0	62	85	Heat shock protein 17.4
miR775	AT1G01040.1	TTCGATGTCTAGCAGTGCCT	UGGAACUGCUAGACAUAGAG	3.0	3217	3236	CAF, SUS1, SIN1, ASU1, EMB76/60 DCL1
miR829	AT5G18560.1	AGCTCTGATACCAAATGA	UUACCUUGAAGUUUUGAUUUG	1.5	1284	1304	AP2 domain-containing transcription factor
miR840	AT5G55930.1	TTGTTTAGGTCCCTTAGTTTCT	AAGUUAAGGGACUCAAACAA	3.0	2623	2642	Oligopeptide transporter 1
miR850	AT2G43240.1	AAGATCCGGACTACAACAAAGC	CCUUGUUCUAGUUCGGAUUUU	3.0	129	148	Nucleotide-sugar transporter

**Expectation**: value assigned to the alignment of the mature miRNA and the target. The value ranges from 0 (perfect alignment) to 5; **Target start**: the base position where the annealing with the miRNA starts; **Target end**: the base position where the annealing with the miRNA ends.

### Expression profiling of miRNAs identified in *A. thaliana* leaf and callus tissues

**Figure 3:**
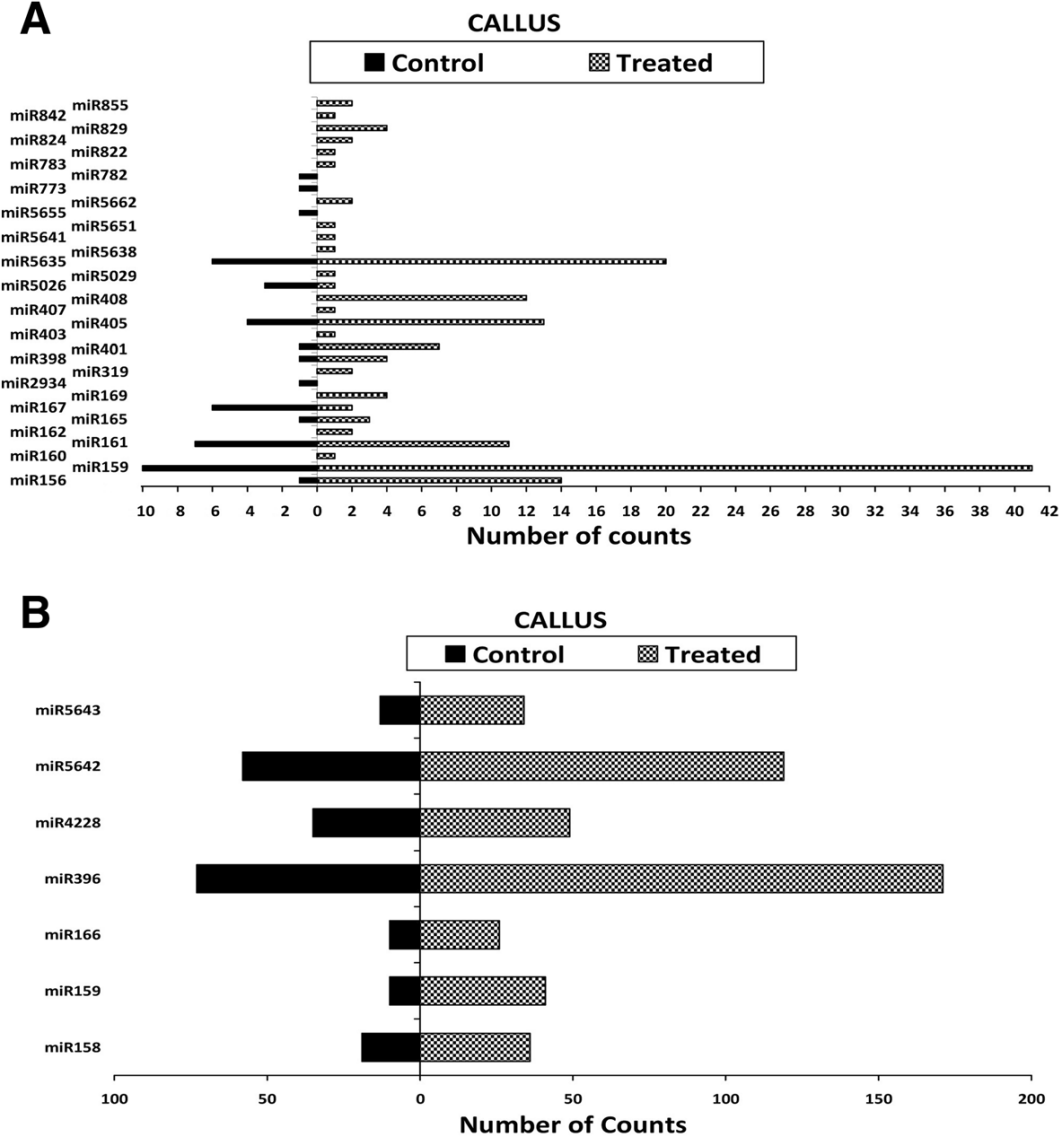
**Expression profiling of miRNA identified in** ***A. thaliana*** **callus tissue using Illumina technology.** Expression of miRNA with **(A)** low counts and **(B)** high counts respectively.

**Figure 4:**
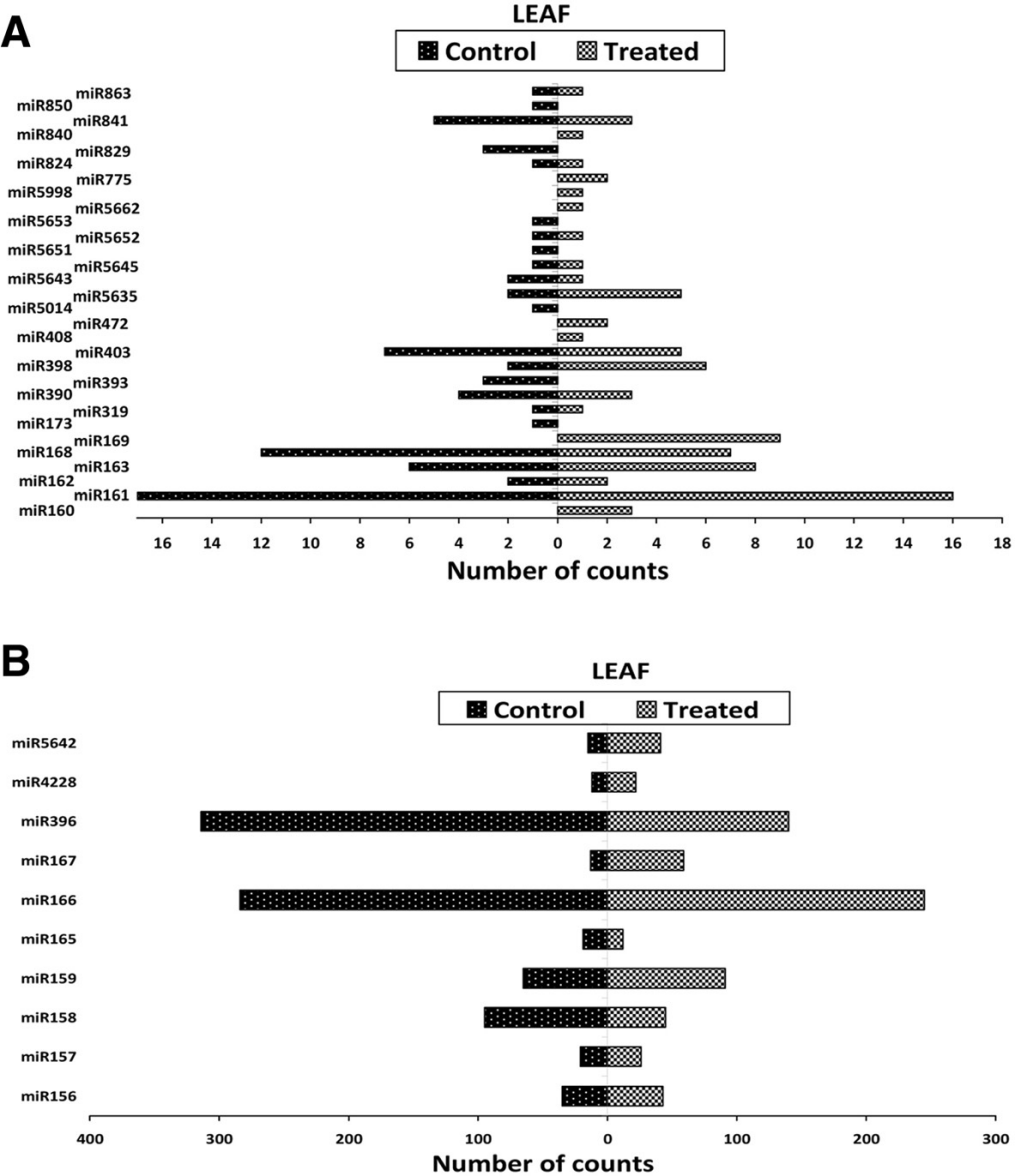
**Expression profiling of miRNA identified in** ***A. thaliana*** **leaf tissue using Illumina technology.** Expression of miRNA with **(A)** low counts and **(B)** high counts respectively.

H-T sequencing is an efficient tool to identify miRNAs and accurately measure their expression profiles especially those with low expression levels, in plants [38,39]. The expression profiles of each miRNA obtained from the sequencing and expressed by read counts in each library vary from 0 to 171 in the callus tissues (Figure 3A, B) and from 0 to 314 in the leaf tissues (Figure 4A, B). The regulation was observed for each miRNA where the log_2_ ratio of normalized expression under treatment was greater than 1 or less than −1 [34,39]. Eleven miRNAs were up-regulated with a log_2_ fold change range between 1.1 and 3.9 in the callus tissues (Table 1) and four miRNAs with a log_2_ fold change range between 1.3 and 2.2 in the leaf tissues (Table 2). The expression of two miRNAs was down-regulated with a log_2_ fold change less than −1 in both callus and leaf tissues. In the callus tissues, 18 miRNAs were only expressed in the treated library; four miRNAs in the control library and fourteen miRNAs had similar expression in the two libraries with a log_2_ fold change range between −1 and 1 (Table 1). In the leaf tissues, 8 miRNAs were only expressed in the treated library; seven miRNAs in the control library and nineteen miRNAs had similar expression in the two libraries (Table 2). The most differentially expressed miRNA with a highest fold change in the callus tissue was miR156 and in leaf tissue, miR167.

### Quantitative miRNA expression analysis by real time PCR

**Figure 5:**
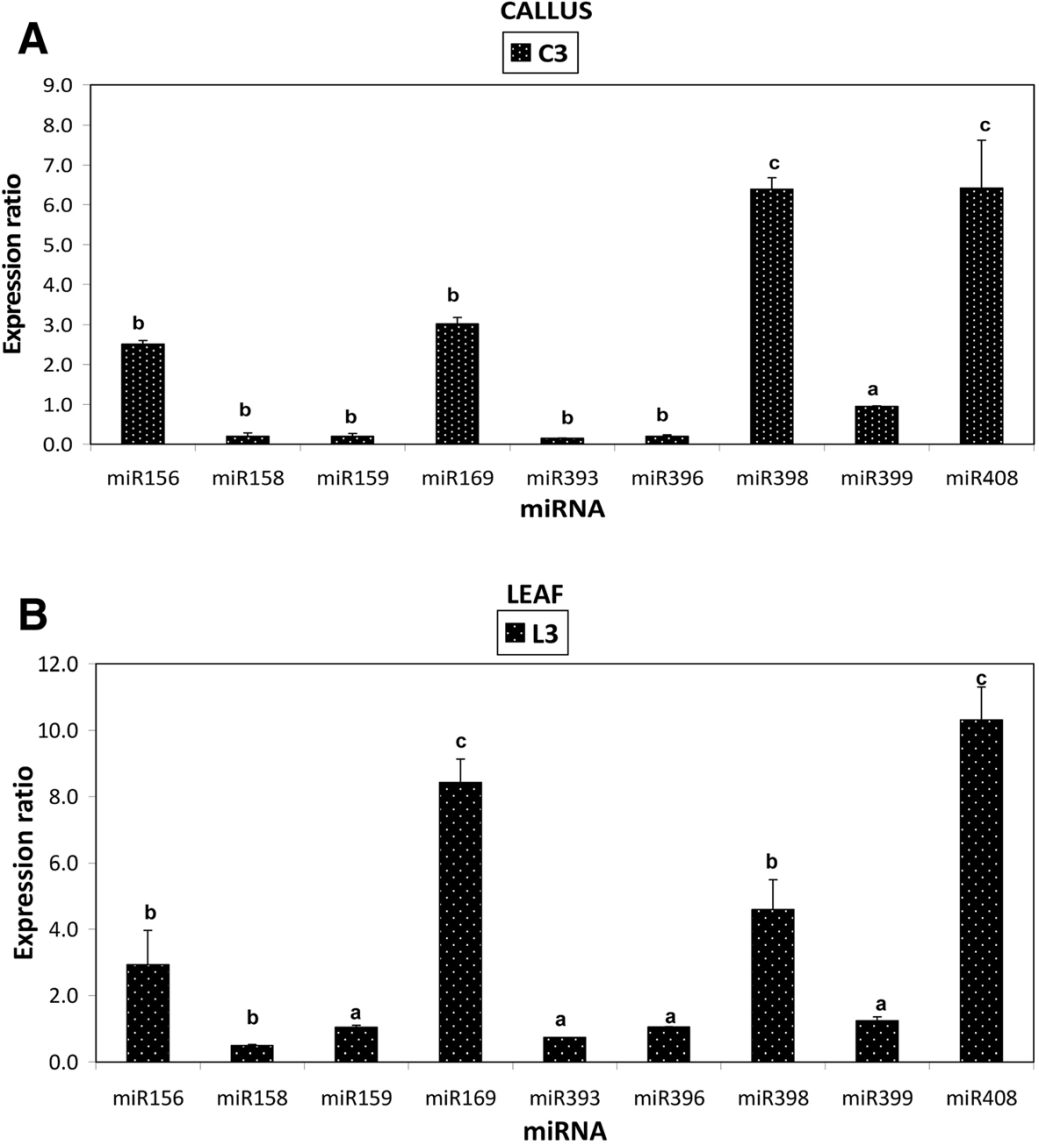
**qPCR of miRNA expression analysis in LPS-elicited callus tissue (A) and leaf tissue (B).** Treated samples (C3 and L3) showed differential gene expression relative to control samples (C0 and L0 respectively). The data was normalized using U6 small nuclear RNA to give the relative gene expression wherein error bars represent the standard error of mean. Expression analysis was performed on three biological repeats with three technical replicates of each. (a) Indicates no significant differences, with P > 0.05, (b) indicates that there was a significant difference with P < 0.05, (c) indicates that there was a highly significant difference with P < 0.01.

Expression analyses of nine miRNAs were conducted at 0 and 3 h post induction to validate if the sequencing data reflected their expression. This was normalized against the U6 small nuclear RNA to give the relative expression (Figure 5A, B). The expression data was then compared against the H-T sequencing data analysis which revealed that five (miR156, miR169, miR398, miR399 and miR408) of the nine miRNAs in callus tissue and six (miR158, miR159, miR169, miR393, miR396 and miR408) of the nine miRNAs in leaf tissue showed expression patterns that were similar to those observed with the H-T sequencing data. In both callus and leaf tissues, four miRNAs (miR156, miR169, miR398 and miR408) were up-regulated, two miRNAs (miR158, and miR393) were down-regulated with two other miRNAs (miR159 and miR396) only found in the callus tissue (Figure 5A, B). Furthermore, in the callus tissue, miR399 and three miRNAs in the leaf tissue (miR159, miR396 and miR399) were not differentially expressed between the untreated and treated samples. The qPCR showed that miR393 was expressed but significantly down-regulated in the treated callus tissue which contrasted the results obtained by sequencing analysis, which indicated that it was not expressed in the treated callus tissue. A similar observation was done for miR399 in the leaf tissue. In callus and leaf tissues, miR408 showed the highest relative expression contrary to the sequencing analysis which indicated that the most abundant up-regulated miRNAs was miR156. The greatest degree of down-regulation in response to LPS was shown by miR393 in the callus tissue.

### Expression analysis of miRNA target genes by real time PCR

**Figure 6:**
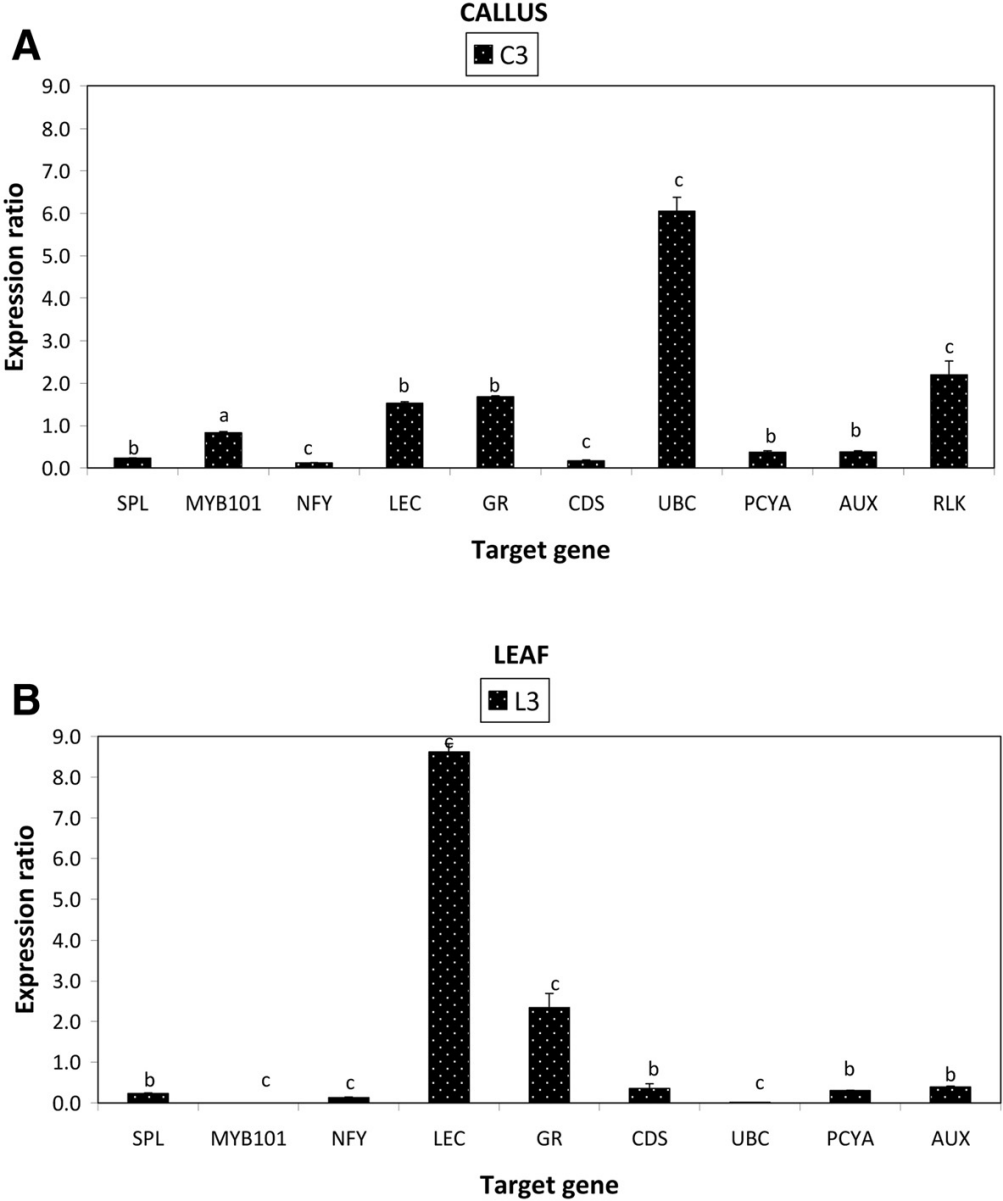
**qPCR of miRNA target gene expression analysis in LPS-elicited callus tissue (A) and leaf tissue (B).** Treated samples (C3 and L3) showed differential gene expression relative to control samples (C0 and L0 respectively). The data was normalized using *elongation factor 1-alpha* and *actin 8* to give the relative gene expression wherein error bars represent the standard error of mean. Expression analysis was performed on three biological repeats with three technical replicates of each. (a) Indicates no significant differences, with P > 0.05, (b) indicates that there was a significant difference with P < 0.05, (c) indicates that there was a highly significant difference with P < 0.01. Auxin response factor 10 (AUX), Concanavalin A-like lectin protein kinase (LEC), Copper superoxide dismutase (CDS), Nuclear factor Y subunit A8 (NFY), Growth regulating factor 4 (GR), Myb domain protein 101 (MYB101), Plantacyanin (PCYA), Receptor-like protein kinase (RLK), Ubiquitin-protein ligase (UBC) and Squamosa promoter-binding-like protein (SPL).

To evaluate the correlations between miRNA expression profiles and their target genes, we performed quantitative expression analysis of 10 corresponding target genes of the miRNAs studied in the above section (Figure 6A, B). In the callus tissue, the expression profiles of 8 target genes (*auxin response factor 10, concanavalin A-like lectin protein kinase, copper superoxide dismutase, nuclear factor Y, Myb domain protein 101, plantacyanin, receptor-like protein kinase,* and *squamosa promoter-binding-like protein*) behaved as expected (Figure 6A), i.e. if miRNA expression was up-regulated/induced then target gene expression was down regulated/repressed and vice versa. In the leaf tissue the expression profiles of six target genes (*auxin response factor 10, concanavalin A-like lectin protein kinase, copper superoxide dismutase, nuclear factor Y, growth regulating factor 4* and *plantacyanin*) behaved as expected (Figure 6B). In the callus tissue, in five cases (*concanavalin A-like lectin protein kinase, copper superoxide dismutase, nuclear factor Y subunit A8, squamosa promoter-binding-like protein* and *plantacyanin*) out of eight cases of expected expression profiles, where the miRNA profiles had a significant p value either p < 0.05 or p < 0.01, the expected trend in the profile of the target gene was also significant with p value either p < 0.05 or p < 0.01. Similar observations were made in the leaf tissue with three cases (*copper superoxide dismutase, nuclear factor Y* and *plantacyanin*) out of six exhibiting the expected expression profile. In the callus tissue two miRNAs (miR396 and miR399) and their corresponding target genes (*growth regulating factor 4* and *ubiquitin-protein ligase* respectively) showed no agreement in their expression profile as expected. This was also the case in the leaf tissue for miR156, miR159, miR399 with their corresponding target genes; *squamosa promoter-binding-like protein, Myb domain protein 101* and *ubiquitin-protein ligase* respectively.

## Discussion

Current indications predict a multifaceted role for miRNAs in plant innate immune responses, from pathogen recognition to modulating hormone responses and coordinating expression of defense genes [9,10,27]. miRNAs can act as both positive and negative regulators of plant immune responses, either alone or in combination with regulatory proteins where they contribute to key regulatory checkpoints modulating both MTI and ETI [26]. However, the networks involved in miRNAs, mRNA and plant hormone signaling is still unclear. It has been noticed that different miRNAs can target the same gene but their expression pattern varies with the type of plant and pathogen under study [40]. In addition, it is unknown whether or not miRNAs function the same way *in vivo* because the expression pattern, timing, and cellular location may differ among miRNAs and their targets.

Although miRNA biogenesis is important for establishment of MTI, miRNA species involved in this process have not been fully explored [26]. Here, small RNA sequencing was done to obtain an overview of the effect of LPS elicitation on the microtranscriptome of *A. thaliana* leaf and callus tissues. In addition, some of the effects were further investigated and verified by the more sensitive qPCR technique [10]. It was reported (and also observed in this study) that H-T sequencing data and qPCR-based assays may give different measures of the same transcript in the same biological sample. Like-wise, it can happen that two miRNAs with similar numbers of sequencing reads may in fact differ substantially in their absolute abundances in a sample [41]. miRNAs have also been shown to have variable expression patterns with regard to tissue differentiation and developmental stages [11].

In this study, a total of 630 highly conserved plant miRNAs were identified in both callus and leaf tissues of Arabidopsis. Some of the stress-responsive miRNA families are deeply conserved among various plant species [42-44]. The class of small RNAs of 24 nucleotides was the most abundant class of miRNA identified (Figures 1A and 2A), consistent with previous findings where small RNAs of 24 nucleotides were predominant in plant microtranscriptomes [37,42,44,45]. In addition to small RNA sequencing and identification, Illumina sequencing technology as performed in previous reports [5,36], also measured the expression patterns of each identified miRNA in response to LPS (Figure 5A, B). The deep coverage of mature miRNAs obtained allowed us to compare the normalized number of counts of each miRNA in a treated library to that in the untreated library to find miRNAs that were up-regulated or down-regulated (Tables 1 and 2). Most of the identified miRNAs from treated samples exhibited higher expression compared to the untreated samples, revealing evidence of the effect of LPS on the microtranscriptome of *A. thaliana* leaf and callus tissues.

miRNAs are critical key regulators of gene expression as they respond rapidly to stress by regulating the existing pool of mRNAs [9]. Their putative targets were predicted using the web-based psRNATarget program (Tables 3 and 4). The identification of target mRNAs, together with the significance of their regulation by miRNAs, are key contributors to understanding the biological response. Previous studies showed that miRNAs induced under stress conditions are generally expected to target negative regulators of stress responses or positive regulators of processes inhibited by stresses. Moreover, miRNAs down-regulated by stress are predicted to repress the expression of stress-inducible genes and/or positive regulators [46]. In this study, the major group of predicted target genes are TFs, themselves controllers of gene expression. Some of those predicted TFs (Squamosa promoter binding protein-like 2, Myb domain protein 101, Homeobox-leucine zipper family protein, CCAAT-binding TF (CBF-B/NF-YA), etc.) are regulated by the identified **miR156, miR159, miR165, miR166, miR169, miR319, miR408, miR829, miR2934, miR5029** and **miR5642** (Tables 3 and 4).

The sequencing revealed that **miR156** was up-regulated with a 3.9 fold change in the treated callus tissue and without any expression change in the leaf tissue (Tables 1 and 2). The expression profile was validated with the qPCR which indicated an up-regulation in both callus and leaf tissues with a fold change of 2.5 and 2.9 (Figure 5A, B). A target gene for this miRNA encodes squamosa promoter binding like protein (SPL). The SPL gene family belongs to a group of plant-specific zinc finger protein genes that encodes TFs known to be involved in responses to abiotic - and biotic stresses, and the activation of other TFs [47,48]. The expression profile of SPL measured by qPCR showed a significant down-regulation in both callus and leaf tissues (Figure 6A, B). This indicates that the up-regulation of miR156, leading to lower levels of SPL, would enhance the *A. thaliana* response to LPS.

The H-T sequencing showed that the expression of **miR159** was up-regulated in the treated callus tissue and without any expression change in the leaf tissue (Tables 1 and 2). qPCR validated the expression observed in the leaf tissue but a contrasting down-regulation was found in the callus tissue (Figure 5A, B). miR159 primarily regulate signal transduction and development of plants under various stress conditions [7]. The target for this miRNA encodes Myb domain protein 101 (Myb 101) which was shown by qPCR to be down-regulated in both tissues (Figure 6A, B). This observation led us to consider the expression profile of miR159 revealed by the H-T sequencing result rather than the one revealed by the qPCR. A previous study by Reyes and Chua [49] found miR159 also to be induced in Arabidopsis in response to infection with *Pseudomonas syringae.* Consequently, miR159 mediates cleavage of Myb33 and Myb101 transcripts, which encode positive regulators of abscisic acid (ABA) responses. This was hypothesised to activate the salicylic acid (SA) signaling pathway in turn, to promote SA-mediated defense responses [49,50]. Similarly, our data could imply that miR159 influences hormone signaling pathways to trigger defense responses to LPS but this need to be confirmed with further investigation.

The similar down-regulated pattern observed for SPL and Myb101 could be correlated to the co-expression network study of Wang *et al.* [48] who demonstrated that SPL genes can activate other TF families (B3, bZIP, WRKY, MYB, bHLH, and MADs-box) and form a complex control network. In line with previous findings on stress-regulated miRNAs in Arabidopsis and rice [17,34], the up-regulated expression of miR156 and miR159 may lead to the repression of their predicted target TFs which would lead to the activation of defense pathways in response to LPS perception.

**miR169** was induced in both callus and leaf tissues as revealed by the H-T sequencing results (Tables 1 and 2) and the expression profile was validated by qPCR which showed significant up-regulation in both tissues (Figure 5A, B). miR169 expression is induced in rice and Arabidopsis under drought [51] and salt stress [52] and down-regulation of their target genes resulted in tolerance to these environmental stresses. The target for this miRNA encodes the nuclear factor Y family (NF-Y), a group of TFs that have three distinct subunits (NF-YA, NF-YB, and NF-YC) that bind to the CCAAT box [53,54]. The qPCR revealed that in both tissues the expression of the nuclear factor Y family transcript was significantly down-regulated (Figure 6A, B). Based on these reports and from the qPCR results, the up-regulation of miR169, by decreasing the levels of the nuclear factor Y family, might contribute to the LPS-induced responses in *A. thaliana* since the down-regulation of some genes could also be very important for plants to overcome abiotic/biotic stresses.

The **miR158** was down-regulated due to LPS elicitation in the leaf tissue and was not differentially expressed in the callus tissue according to the H-T sequencing result (Tables 1 and 2). qPCR revealed that it was significantly down-regulated in both tissues (Figure 5A, B). miR158 targets many genes which encode pentatricopeptide repeat (PPR) family proteins, as well as fucosyl transferase genes encoding glycosyl transferases for cell wall xyloglucan biosynthesis. PPRs are putative RNA binding proteins involved in RNA processing, metabolism, editing or translation [22]. Although the fact that their function in plant pathogen resistance remains to be explored [22], the down-regulation of their regulated miRNA, may have contributed to increase the response of *A. thaliana* triggered by LPS.

Another category of stress-responsive miRNAs identified in this study is **miR160** which was shown to be induced in both callus and leaf tissues following LPS treatment (Tables 1 and 2). miRNA160 was reported to positively induce Flg22 induced callose deposition [26]. Furthermore, miR160 was highly induced in Arabidopsis leaves collected at 1 and 3 h post-inoculation with the *hrcC* mutant of *P. syringae* pv. tomato, DC3000 [42]. miR160 regulates genes involved in the auxin signaling pathway, including auxin response factors and auxin receptors. ARFs are known to bind auxin-response elements and either activate or repress gene expression [7]. The qPCR revealed that in both tissues the expression of ARF 10 was significantly down-regulated (Figure 6A, B). Many biotrophic pathogens can synthesize auxin or auxin-like molecules to promote virulence. As a result, host plants have developed several counter measures, including miRNA-mediated gene regulation, to suppress auxin signaling and subsequently inhibit pathogen growth [24,55]. Our results imply that the up-regulation of miR160, by decreasing the levels of ARF 10, might contribute to enhance *A. thaliana* responses induced by LPS.

The expression profile of **miR393** was repressed in the leaf tissue as shown by the H-T sequencing results (Table 2) but no single read was detected in the callus tissue. However, the more sensitive qPCR confirmed the expression profile of miR393 by revealing a slight down-regulation in the leaf tissue and a significant down-regulation of 6.6 fold in the callus tissue (Figure 5A, B). This observation was in contrast with the study of Navarro *et al.* [24] who reported that Arabidopsis miR393 expression was induced with a two fold increase following treatment with flg22. This might be due to the different chemical structures of flg22 *vs*. LPS and different mechanisms of perception. miR393 was reported to regulate auxin signaling and defense response by targeting TIR1, (part of the ubiquitin ligase complex SCFTIR1) which represses auxin signaling and enhances bacterial disease resistance [22,24]. In this study, target prediction revealed that miR393 targets genes which encode concanavalin A-like lectin protein kinase family proteins. The qPCR indicated, with the expected trend in the expression profile, that the concanavalin A-like lectin protein kinase was significantly up-regulated in both tissues with a 8.6 fold change in the leaf tissue (Figure 6A, B). In addition, the lectin receptor-like protein kinase (LecRLK) (At3G59740.1), only quantified by qPCR in the callus tissue, showed a significant up-regulation (Figure 3C). In addition to plant growth and development, LecRLKs play crucial roles in adaptive responses to various abiotic and biotic stressors [56], and might act as inducible receptors for recognition of extracellular carbohydrate-based MAMPs like LPS [57]. The down-regulation of miR393 and up-regulation of the corresponding LecRLK target gene indicates that this could be part of enhancing the perception capabilities of *A. thaliana* cells exposed to LPS.

Another LPS-responsive, stress-regulated miRNA identified in this study is **miR396,** known to target growth regulating factors (GRFs) [58,59]. miR396 was up-regulated in the callus tissue and down-regulated in the leaf tissue according to the H-T sequencing results (Tables 1 and 2). In contrast, qPCR revealed a down-regulation of miR396 in the callus tissue and no differential expression in the leaf tissue (Figure 5A, B). Tissue differentiation [11] could explain this variation in the expression profile of miR396 in the two tissue types. Consequently, the qPCR showed that the GRF-4, target of miR396, was significantly up-regulated in both callus and leaf tissues (Figure 6A, B). These results correlated with the expression patterns of miR396 given by the qPCR in the callus tissue and H-T sequencing result obtained in the leaf tissue. In this regard, the increase of the expression of the GRF-4 by the reduction of miR396 expression suggests the involvement of this GRF in the *A. thaliana* response to the LPS elicitation. This is supported by recent data that the GRF TFs are also involved in biotic stress responses and may play a role in coordinating the interaction between developmental processes and defense dynamics [59].

The H-T sequencing results showed that **miR399** was only expressed in the callus tissue (Table 1) but the qPCR revealed that it was not differentially expressed in both tissues (Figure 5A, B). A putative target of miR399 encodes ubiquitin-protein ligase [60]. The qPCR of ubiquitin-protein ligase showed complete repression in the leaf tissue and an up-regulation in the callus tissue (Figure 6A, B). During plant-pathogen interactions proteins that function as negative regulators of defense are targeted and degraded [61,62]. Since ubiquitin-protein ligase plays a role in this process [62], our data suggests that it may be similarly involved in the response of *A. thaliana* cells towards LPS.

We found that **miR398**, proposed to be directly linked to the plant stress regulatory network, was up-regulated in both tissues as shown by the H-T sequencing (Tables 1 and 2) and the expression pattern was validated by the qPCR which revealed a significant up-regulation in both tissues (Figure 5A, B). In Arabidopsis, prior studies demonstrated that miR398 is involved in responses to abiotic - and biotic stresses and it targets at least four mRNAs which include the cytosolic copper/zinc superoxide dismutase1 (CSD1), the chloroplastic CSD2, a subunit of the mitochondrial cytochrome C oxidase, COX5b-1, and the copper chaperone for superoxide dismutase [63,64]. The significant down-regulation of CSD revealed by the qPCR in both tissues (Figure 6A, B) indicated a role for miR398-mediated gene regulation in response to LPS. CSDs limits the formation of reactive oxygen species (ROS) and institute their removal. During plants’ early response to pathogen invasion, ROS are required to trigger the overall response system that includes the hypersensitive response and defense gene activation [65]. The repression of the CSD by the overexpression of miR398 might thus be required to enhance *A. thaliana’s* response to LPS perception.

Similarly, **miR408** was induced in both callus and leaf tissues as revealed by the H-T sequencing (Table 1 and 2) and the expression profile was validated by the qPCR which showed that it was significantly up-regulated in both tissues (Figure 5A, B). miR408 has been reported as a negative regulator of plantacyanins [66] and the qPCR of plantacyanin showed a significant down-regulation in both tissues (Figure 6A, B). Plantacyanins (blue copper proteins) have been proposed to function in cell-to-cell signaling, stress responses and involved in redox reactions occurring during primary defense responses [67], but functional characterization is hindered by the complexity of redox processes in biological systems. The regulation of genes encoding copper proteins by miR398 and miR408 suggests a link between copper homeostasis and its contribution to the activation of the *A. thaliana* response to LPS through mechanisms that are as yet unknown.

In addition, the sequencing results also revealed that various other stress-regulated miRNAs were expressed in response to LPS which include: **miR161, miR165, miR166, miR167, miR168 miR401, miR403, miR405 and miR5635**. These have previously been found to be regulated in *A. thaliana* seedlings exposed to cold stress, dehydration, high salinity, nitrogen deficiency and the stress hormone abscisic acid [17,46,68]; and in other plants such as rice, Populus and tobacco [20,21,69].

## Conclusions

**Figure 7:**
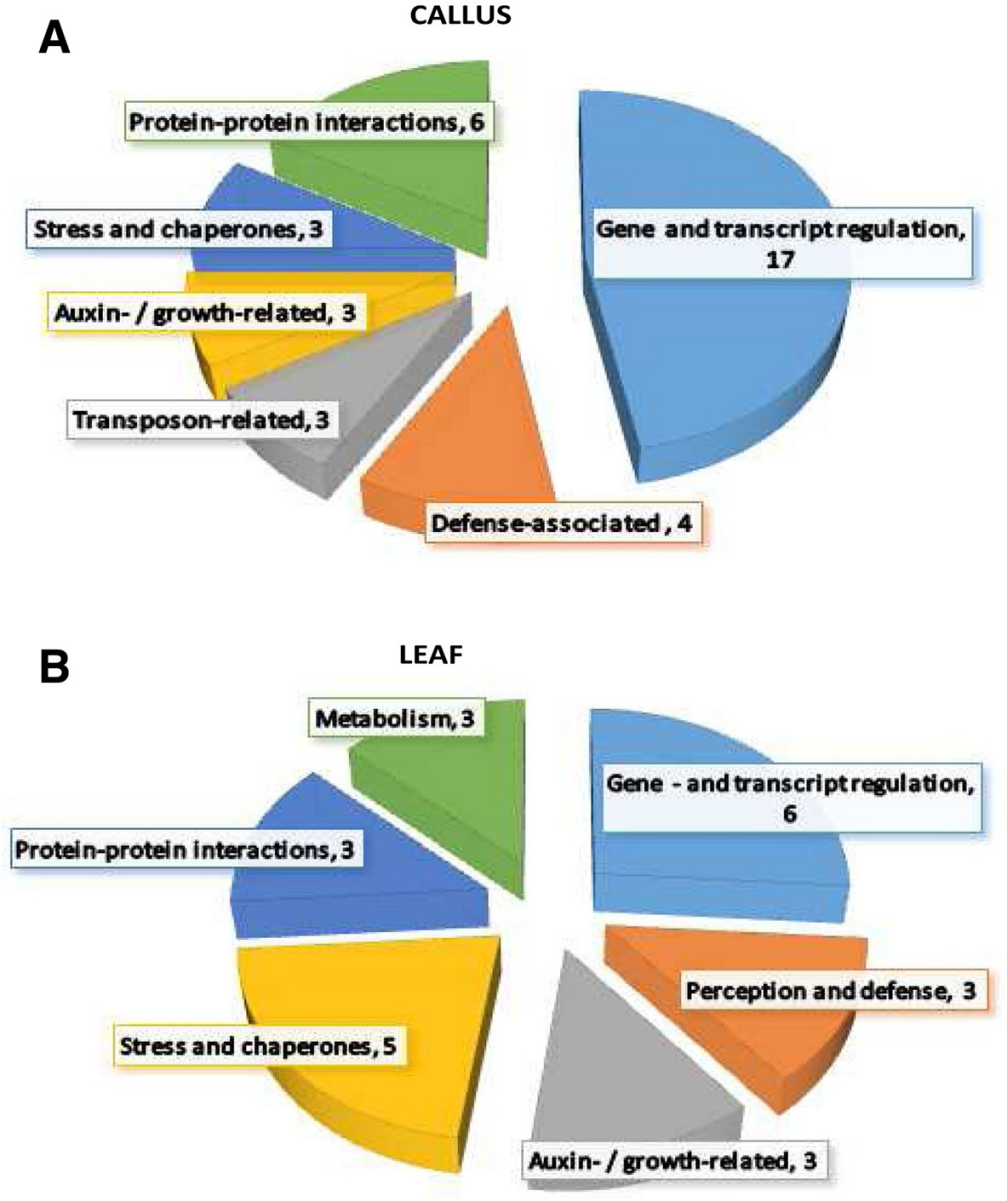
**Comparative charts showing functional categories of the predicted target genes of miRNAs differentially expressed in callus tissue (A) and leaf tissue (B) responding to LPS elicitation.** Each target gene was grouped in their corresponding biological class and the number of genes per category is indicated. The charts show the similarities and slight diffferences between undifferentiated **(A)** and differentiated **(B)** systems.

Multifaceted roles for miRNAs as molecular regulators during plant immune – and defense responses have been proposed [10,27]. Illumina H-T sequencing technology and qRT-PCR allowed us to gain a global perspective of the expression profiles of miRNAs in *A. thaliana* leaf and callus tissues following the perception of LPS. In callus tissues 358 miRNAs, belonging to 49 miRNA families and in leaf tissues, 272 miRNAs belonging to 40 miRNA families were identified and their target genes predicted. The results revealed evidence of the effect of LPS on the microtranscriptome of *A. thaliana* leaf and callus tissues, resulting in dynamic changes and differential re-programming (summarised in Figures 7A, B). Together with the effects on their corresponding target genes, this indicates some of the early events leading up to MTI.

These observations add insights to our previous contributions regarding LPS as a trigger of the expression of a broad range of defense-associated genes in *A. thaliana* [32,33,70-72]. The findings presented here reflect a novel view of LPS as a potent MAMP and potential plant-priming agent, revealing that *A. thaliana* exhibit a molecular system for recognition and sensing LPS [57] which in turn differentially regulate a subset of stress-regulated miRNAs. Taken together, all of the LPS-responsive miRNAs target several stress-related genes, including some encoding TFs and signal generating proteins, at one time. In turn, each target gene is potentially involved in the regulation of downstream biochemical processes, implying regulation and crosstalk of gene expression.

Our study thus provides valuable information to understand the function of miRNAs in the regulation of plant responses to a biotic stress in general and LPS/MAMP perception in particular. The results also contribute significantly to increase the knowledge about how miRNAs are utilised to reprogram cellular metabolism upon perception of MAMPs during pathogen attack.

## Methods

### Plant material and LPS preparation

Extraction and purification of LPS from *Burkholderia cepacia* were done as previously described using the hot phenol method [31]. Purified LPS, treated with RNase, DNase and proteinase K, was solubilised in half-strength Murashige and Skoog (MS) media for cell treatment or 10 mM MgCl_2_ for leaf treatment [71,72]. *A. thaliana* ecotype Columbia 0 callus cells were grown on solid agar gel medium containing MS salts including 3% sucrose (w/v), 0.8% agar (w/v), 1.0 mg L^−1^ 2,4-dichlorophenoxyacetic acid, 100 mg L^−1^ myo-inositol and B5 vitamins [71]. Five week old *A. thaliana* plants grown in a controlled greenhouse environment under a 10/14 h light–dark photoperiod [70] were used for experiments.

### Treatment of *Arabidopsis thaliana* leaf - and callus tissues

*A. thaliana* leaves were pressure infiltrated with a blunt ended syringe containing 100 μg mL^−1^ dissolved in 10 mM MgCl_2_. Plants were allowed to stand at room temperature and the leaves were harvested after 0 and 3 h (control group = L0 and treated group = L3) for subsequent experiments. Friable callus cells were transferred to liquid half-strength MS medium containing 100 μg mL^−1^ LPS (2 g in 10 mL solution) and placed on an orbital shaker at 100 rpm and 24°C. Cells were harvested at 0 and 3 h (control group = C0 and treated group = C3) by means of a vacuum filtration system (Millipore, Billerica, MA, USA). For the control and treated group, five plants were used for each group.

### microRNA isolation

miRNA was isolated from the callus tissues and leaves using a high pure miRNA isolation Kit (Roche, Mannheim, Germany). Briefly, 100 mg of frozen leaf tissue were ground with a mortar and a pestle in the presence of liquid nitrogen. The resulting powder was transferred to sterile tube containing 400 μL of 20% binding buffer and centrifuged for 2 min at maximum speed. To 150 μL of lysate, 312 μL of binding buffer was added, briefly vortexed, and floowed with 200 μL binding enhancer. The total mixture was pipetted into the high pure filter tube, centrifuged for 30 s at 13 000 xg and the flow through was discarded. After this 500 μL of wash buffer working solution was added, centrifuged for 30 s at 13 000 xg and the flow through was discarded. This step was repeated with 300 μL of wash buffer working solution followed by centrifugation at 13 000 xg for 1 min in order to dry the filter fleece completely. Finally 100 μL elution buffer was added and after 1 min of incubation, miRNAs were eluted after centrifuging for 1 min at 13 000 xg.

The miRNA yield was measured with a ND-1000 spectrophotometer (NanoDrop, Wilmington, DE, USA) and the integrity and size distribution of the isolated miRNAs were checked electrophoretically on a denaturing gel (15% acrylamide/TBE/urea) and the visualization was done by ethidium bromide staining under UV light.

### cDNA library construction and sequencing of the miRNA

The small RNA libraries construction (control and treated after 3 h) and Illumina sequencing were performed using the Illumina MiSeq system (Inqaba Biotech, Pretoria, South Africa). Briefly, the quality and quantity assessment for each isolated miRNA sample were done using the Bioanalyzer (fragment distribution) and fluorometer. Before constructing the miRNA libraries, the miRNA were ligated at both ends with 3' and 5' adapters and the products used for cDNA synthesis and PCR-amplification using the TruSeq Small RNA sample PrepKit following the supplier’s instructions (Illumina Inc., San Diego, CA, USA). Finally the amplified PCR products after gel purification and recovery using the TruSeq Small RNA Sample PrepKit were subjected to the MiSeq system for deep sequencing.

### Bioinformatic analysis, miRNA identification and target prediction

All sequencing raw reads of the four miRNA libraries (2 for the callus tissues and 2 for the leaf tissues) were cleaned of sequence adapters; low quality tags and reads shorter than 15 nucleotides or longer than 55 nucleotides. The transcripts were mapped to the *A. thaliana* genome and miRBase release 20.0 (http://www.mirbase.org) databases with default parameters. Alignment results were processed to identify miRNA sequences that corresponded exactly in size and nucleotide composition to reported plant miRNA sequences. Expression of each miRNA was evaluated as a number of counts in each library. Target prediction of each miRNA was by psRNATarget (http://plantgrn.noble.org/psRNATarget/), miRTarBase: the experimentally validated microRNA-target interactions database (http://mirtarbase.mbc.nctu.edu.tw/), PMRD: plant microRNA database (http://bioinformatics.cau.edu.cn/PMRD/), Next-Gen sequence databases (https://mpss.udel.edu/index.php) and AthaMap (http://www.athamap.de/), based on complementarity scoring and secondary structure analysis [10,35,37,73-75]. All additional bioinformatic analyses were performed using the CLC Genomic workbench 6 software (CLC Bio, Cambridge, MA, USA).

### Evaluation of miRNA expression profile by real time PCR

To validate the sequencing results with the bioinformatics-based analysis and based on their key function in gene regulation, the following mature miRNA were selected for expression profile analysis: miR156, mi158, miR159, miR169, miR393, miR396, miR398, miR399 and miR408. To quantify their expression, RNA was isolated from the callus tissues and leaves using a high pure miRNA isolation Kit (Roche, Mannheim, Germany) as described. The isolated RNA samples were polyadenylated and reverse transcribed using the Mir-X miRNA first-strand synthesis Kit (Takara, Clontech, Mountain View, CA, USA). Real time PCR (qPCR) was performed on the Rotor gene-3000A machine (Qiagen, Venlo, Netherlands) using the Mir-X miRNA qRT-PCR SYBR Kits (Takara, Clontech, Mountain View, CA, USA) with the entire sequence of the mature miRNA as the miRNA-specific 5’ primer (10 μM) and the universal reverse primer (mRQ 3’ primer, supplied with the kit). The cycling conditions were as follows: initial denaturation for 10 min at 95°C followed by amplification and quantification cycle repeated 40 times each consisting of 15 s denaturing at 95°C, 20s annealing at primer specific temperatures (Tm), 20 s primer extension at Tm + 2°C. At the end of each PCR reaction, a melting curve was determined, and only samples that displayed a one-peak melting curve at the right annealing temperature were used for subsequent analysis. The miRNA expression was calculated from three biological replicates to ensure statistical rigor. The relative standard curve method was used to quantify the expression and the U6 small nuclear RNA (supplied with the kit) was used as an internal control to normalize the expression levels of mature miRNAs as previously reported [76]. U6 is a class of metabolically stable small non-coding RNAs, with sizes of about 100 nucleotides in length, which are found in the nuclei of eukaryotic cells, showing a high degree of conservation [76]. qPCR data was statistically compared between untreated and treated samples at each time point using one-way ANOVA [77] with the confidence level of all analyses set at 95%, and values with p < 0.05 were considered to be significant. The miRNA-specific 5’ primers used are provided in Additional file 1: Table S1.

### Quantification of the expression of predicted miRNA target genes

To evaluate whether the expression change of miRNAs in response to LPS correlated with differences in the transcripts from their target genes, we analyzed the expression levels of 10 predicted miRNA target genes. Prior to quantification of their expression levels, total RNA were extracted from drained elicited callus tissue (100 mg) and leaf tissues (100 mg) using Trizol reagent (Invitrogen, Carlsbad, CA, USA) followed by DNase treatment using DNase I (Thermo Scientific, Waltham, MA, USA). The DNase-treated RNA were reverse transcribed to cDNA using a RevertAid™ Premium First Strand cDNA synthesis kit (Fermentas, Thermo Scientific, Waltham, MA, USA). The selected miRNA predicted target genes included: *auxin response factor 10, concanavalin A-like lectin protein kinase, copper superoxide dismutase, nuclear factor Y, subunit A8, growth regulating factor 4, Myb domain protein 101, plantacyanin, receptor-like protein kinase, ubiquitin-protein ligase and squamosa promoter-binding-like protein*. These genes were selected for expression profiling based on the fact that they were predicted as the target genes of the above selected miRNAs for qPCR and also because the sequencing analysis demonstrated the expression of their corresponding regulating miRNA. Their primer pairs were designed using the ‘Primer Quest’ tool (Integrated DNA Technologies, Coralville, IA, USA) from sequences obtained in the data base (Additional file 1: Table S1). qPCR was performed to analyze the expression of each gene on the Rotor gene-3000A instrument (Qiagen, Venlo, Netherlands) using the SensiFAST SYBR No-ROX Kit (Bioline, London, UK). Three biological replicates were used with three technical replicates of each. The cycling conditions were as follows: initial denaturation for 2 min at 95°C followed by amplification and quantification cycle repeated 40 times each consisting of 5 s denaturing at 95°C, 10 s annealing at primer specific temperatures, 20 s extension at 72°C. Quantification of the relative changes in gene expression was performed as described above with *elongation factor 1-alpha* and *actin 8* as references genes.

### Availability of supporting data

The data sets supporting the results of this article are included within the article and its Additional file 1: Table S2.

## Additional file

Additional file 1:
**Table S1.** Base composition of primers designed for selected miRNAs and target genes for qPCR. **Table S2.** Summary of Illumina high-throughput sequencing of *Arabidopsis thaliana* callus and leaves tissues untreated and treated with lipopolysaccharides.

## Abbreviations

ABA: Absiscic acid
ARF: Auxin response factor
CSD: Copper/zinc superoxide dismutase
DCL: Dicer-like
ETI: Effector-triggered immunity
GRF: Growth regulating factor
H-T: High-throughput
LecRLK: Lectin receptor-like kinase
LPS: Lipopolysaccharide
MAMP: Microbe-associated molecular pattern
miRNA: Micro RNA
MTI: MAMP-triggered immunity
NF-Y: Nuclear factor Y
RISC: RNA-induced silencing complex
ROS: Reactive oxygen species
SA: Salicylic acid
SPL: Squamosa promoter binding like protein
TF: Transcription factor
