# Supplementary material for: Lipopolysaccharide perception leads to dynamic alterations in the microtranscriptome of Arabidopsis thaliana cells and leaf tissues
Source: BMC Plant Biol. 2015 Mar 7;15:79. doi: 10.1186/s12870-015-0465-x (PMC4354979; doi:10.1186/s12870-015-0465-x)
Supplement: Additional file 1: — Table S1. Base composition of primers designed for selected miRNAs and target genes for qPCR. Table S2. Summary of Illumina high-throughput sequencing of Arabidopsis thaliana callus and leaves tissues untreated and treated with lipopolysaccharides. [file 12870_2015_465_MOESM1_ESM.docx]

**Table S1:** Base composition of primers designed for selected miRNAs and target genes for qPCR

| **Target Genes** | **Primer Sequence (5’-3’)** | **Direction** | **Product length** |
| --- | --- | --- | --- |
| Auxin response factor 17 | \| CATCAACCACCGTGAAGTAG \| \| --- \| \| CGTGACCTTGTGGAAAGTAG \| | Forward  Reverse | 107 |
| Squamosa promoter-binding-like protein | \| CGTAAGCCACAAGGAGTATTT \| \| --- \| \| TTCTTCAGATCTCGCGTTAAG \| | Forward  Reverse | 108 |
| Myb domain protein 101 | \| AATCATCTCCGGCCAAATC \| \| --- \| \| CAGGTAACTGAGAAGCCATAC \| | Forward  Reverse | 118 |
| Nuclear factor Y | \| AACGGAGGAAGAGGAAGAA \| \| --- \| \| GTACCAAGCCAAACTCTAACA \| | Forward  Reverse | 109 |
| Concanavalin A-like lectin protein kinase | \| GACATCTAGAGTAGCGGGAA \| \| --- \| \| CCAACCCAAAGGCGTAAA \| | Forward  Reverse | 98 |
| Growth regulating factor 4 | \| CTCTTCAACACCTACCTCATTAC \| \| --- \| \| CACTTCTTACCATCCGTTCTC \| | Forward  Reverse | 100 |
| Copper superoxide dismutase | \| GCGGTAGTGTTCATGGATTTA \| \| --- \| \| ACAGCTATAAACCTGGCAATC \| | Forward  Reverse | 100 |
| Receptor-like protein | \| ATGGGAGAGGCTATCGTAAG \| \| --- \| \| GATCGTAGGAGCCATCAAATC \| | Forward  Reverse | 94 |
| Plantacyanin | \| CCCGAAGGATTATGATGGTTAT \| \| --- \| \| CAGATCGGAGTAGGGAATCT \| | Forward  Reverse | 100 |
| Ubiquitin-protein ligase | \| GAACGAAACAGAGAGAGAGAAG \| \| --- \| \| CCCTTAATCTCCCGAGTCTAT \| | Forward  Reverse | 109 |
| Actin8 | \| CCCAAAAGCCAACAGAGAGA \| \| --- \| \| CATCACCAGAGTCCAACACAAT \| | Forward  Reverse | 140 |
| Elongation Factor 1-α | \| CACCACTGGAGGTTTTGAGG \| \| --- \| \| TGGAGTATTTGGGGGTGGT \| | Forward  Reverse | 137 |
| miR156 | GACAGAAGAGAGTGAGCAC |  |  |
| miR158 | TCCCAAATGTAGACAAAGCA |  |  |
| miR159 | TTTGGATTGAAGGGAGCTCTA |  |  |
| miR169 | CTGGCAAGTTGACCTTGGCTCTGC |  |  |
| miR393 | ATCATGCGATCTCTTTGGAT |  |  |
| miR396 | TTCCACAGCTTTCTTGAACTG |  |  |
| miR398 | TGTGTTCTCAGGTCACCCCTG |  |  |
| miR399 | TGCCAAAGGAGAGTTGCCCTG |  |  |
| miR408 | TGCACTGCCTCTTCCCTGGCT |  |  |

**Table S2:** Summary of Illumina high-throughput sequencing of *Arabidopsis thaliana* callus and leaves tissues untreated and treated with LPS.

| **Samples** | **Raw reads** | **Number of reads after trimming** | **Number of reads after filtering on length** | **Average length**  **after trimming** |
| --- | --- | --- | --- | --- |
| C0 | 1 524 915 | 230 956 | 19 170 | 25.7 |
| C3 | 2 003 307 | 545 876 | 52 963 | 29.7 |
| L0 | 2 018 383 | 212 951 | 18 476 | 23.6 |
| L3 | 2 447 757 | 567 937 | 40 433 | 26.7 |

Untreated callus tissues, C0; treated callus tissues after 3h, C3; untreated leaf tissues, L0 and treated leaf tissues after 3h, L3
